# Method for Data Quality Assessment of Synthetic Industrial Data

**DOI:** 10.3390/s22041608

**Published:** 2022-02-18

**Authors:** László Barna Iantovics, Călin Enăchescu

**Affiliations:** Department of Electrical Engineering and Information Technology, George Emil Palade University of Medicine, Pharmacy, Science and Technology of Targu Mures, 540142 Targu Mures, Romania; calin.enachescu@umfst.ro

**Keywords:** smart sensor, sensor data, smart factory, Industry 4.0, data-quality assessment, prediction problem, predictive power, model fit, classification problem, machine learning, statistical modeling, explainable artificial intelligence

## Abstract

Sometimes it is difficult, or even impossible, to acquire real data from sensors and machines that must be used in research. Such examples are the modern industrial platforms that frequently are reticent to share data. In such situations, the only option is to work with synthetic data obtained by simulation. Regarding simulated data, a limitation could consist in the fact that the data are not appropriate for research, based on poor quality or limited quantity. In such cases, the design of algorithms that are tested on that data does not give credible results. For avoiding such situations, we consider that mathematically grounded data-quality assessments should be designed according to the specific type of problem that must be solved. In this paper, we approach a multivariate type of prediction whose results finally can be used for binary classification. We propose the use of a mathematically grounded data-quality assessment, which includes, among other things, the analysis of predictive power of independent variables used for prediction. We present the assumptions that should be passed by the synthetic data. Different threshold values are established by a human assessor. In the case of research data, if all the assumptions pass, then we can consider that the data are appropriate for research and can be applied by even using other methods for solving the same type of problem. The applied method finally delivers a classification table on which can be applied any indicators of performed classification quality, such as sensitivity, specificity, accuracy, F1 score, area under curve (AUC), receiver operating characteristics (ROC), true skill statistics (TSS) and Kappa coefficient. These indicators’ values offer the possibility of comparison of the results obtained by applying the considered method with results of any other method applied for solving the same type of problem. For evaluation and validation purposes, we performed an experimental case study on a novel synthetic dataset provided by the well-known UCI data repository.

## 1. Introduction

The properties of the data (data distribution, etc.), data quality (missing values, outliers, etc.) and quantity are of utmost importance for the efficient functioning of modern data-driven artificial-intelligence applications that are, most of the time, based on machine learning. Based on the available data, we should chose the most appropriate corresponding analyzing methods. Methods that are not chosen based on data property, quality and quantity could lead to the wrong conclusions. This is why it is so important, as a preliminary step, to analyze the data by using appropriate techniques. This is a step that is skipped frequently in research, leading to the application of less appropriate methods, even when more appropriate methods are available.

Often, it is difficult, or even impossible, to obtain real-life data provided by sensors that should be used for evaluation and testing of algorithms/methods. In such a situation, the feasible option is to work with synthetic data obtained by physical systems or the software simulation of real physical systems. Such an illustrative field, in this sense, is the industry, with recent developments related to Industry 4.0 and smart factories. Motivations of the difficulties to obtain real data provided by sensors for research consists of aspects such as confidentiality of data and industry reticence to share data based on considerations such as industrial secrets and industrial competition. Research performed on synthetic data has advantages versus real data, as such research is cheap and can be generated in the necessary quantity and diversity. One of the limitations of synthetic data consists in the fact that we cannot replicate totally real data based on those applied algorithms, as they could give different results than those applied to real data. Based on this respect, we consider that synthetic data should pass data-quality assessments.

In this paper, we present a mathematical modeling for a data-quality assessment for a specific prediction type of problem-solving. The method is called binary logistic regression (BLR), which is alternatively known as bivariate logistic regression, for the evaluation of predictive power of some predictor (independent) variables, which can be by nominal, ordinal or ratio type on a predicted (dependent) dichotomous variable. The result of the prediction admits a binary classification. For the BLR method, we present the assumptions that must be passed preliminarily for the further applicability of the method. We present an interpretation of the results in a case where all the necessary assumptions are passed, being identified the useful predictor variables and their predicting power. The measure of the predictive power, at the same time, is an indicator of the model fit. It is indicated that the predictive power should belong to at least a minimal class of predicting power that must be established by a human assessor (HA) who has an important central role in the data-quality assessment. Appropriate predictive power indicates that further methods can even be applied.

Based on the predicted probability finally is performed a binary classification whose results are retained in a classification table. Based on the values from the classification table, we can apply any indicators of performed classification quality, such as sensitivity, specificity, accuracy, F1 score, area under curve (AUC), receiver operating characteristics (ROCs), true skill statistics (TSS) and Kappa coefficient. These evaluation results offer the possibility of comparison of the results obtained by applying the BLR with results of any other method that can be applied for performing a binary classification based on the same data.

If the required assumptions fail or the prediction power is low, we can analyze the necessity of the introduction of new predictor variables or the exclusion of redundant predictor variables. It can even be that the available data are not appropriate for research purposes.

BLR is mostly known to be applicable in healthcare research. We would like to notice that frequently is not applied correctly by omitting the verification of the necessary assumptions that should pass to be applicable. In this paper, we treat in-depth the subject of correct application and correct interpretation of the results. Based on this fact this paper represents at the same time a guideline for the correct application of BLR in research to avoid misinterpretations of research data.

For evaluation purposes of the data quality assessment, we have performed an experimental evaluation case study using a recently provided synthetic dataset available on the UCI data repository [1]. We have presented and discussed all the performed steps and interpretation of the results.

The upcoming part of the paper is organized as follows: Section 2 presents a survey on the state-of-the-art data quality assessment methods and presented applications of BLR. In Section 3 the assumptions that must be passed by the BLR method are presented, being treated the interpretation of the results. Section 4 presents an experimental data quality assessment evaluation using a synthetic dataset. Finally, the conclusions are formulated.

## 2. State-of-the-Art Data Quality Assessment

In this section, we present the state-of-the-art regarding the quality assessment of data. Moreover, we present state-of-the-art applications of BLR.

### 2.1. Mathematical Modeling of Data Quality Assessment

The research-data (this is the case among others of synthetic data) property, as well as some other characterizing information, if available, has a significant influence on the appropriateness of the application of specific algorithms. This sometimes could require specific preliminary data analysis and use of available information, hereafter, requiring data-quality assessment. Just to mention a very simple example, for instance, in the case of a search problem in a set of numbers, any times can be applied to the sequential search algorithm. If it is known that the numbers are ordered (the ordering property can be algorithmically verified), then we can apply the binary search algorithm. If it is available, the additional information that the ordered set of numbers will be frequently updated the application in the following of binary search is not appropriate. The motivation consists in the fact that each operation (modification, deletion and adding) should be performed in such a way that the numbers maintain the ordering property, which gives computational complexity if they are frequently executed.

Recently a large effort has been made regarding the development of explainable artificial intelligence (EAI). Applications of EAI include the forecast of climate-change consequences [2] and learning the mental-health impact of COVID-19 in the United States [3]. Matzka [4] presented research performed regarding explainable artificial intelligence applied for predictive maintenance applications on a specific recent dataset available on the UCI data repository [1]. The principal contribution consisted of the design of an explainable model and an explanatory interface. The applied training was performed by using the UCI dataset [1]. Finally, the explanatory performance was evaluated and used for making comparisons. In our research presented in this paper, we used the same dataset [1].

There are diverse approaches in the scientific literature specialized in the quality assessment of different types of data. Wu et al. [5] studied the subject of deraining quality assessment in evaluating some types of images. Ben-Dor et al. [6] performed a quality assessment for several methods, using synthetic imaging spectroscopy data. Dell’Amore et al. [7] assessed the image quality of synthetic aperture radar. Friedrich et al. [8] studied the subjects of data creation and data-quality assessment for databases that are usually used in airports. Papacharalampopoulos et al. [9] performed a deep quality assessment for a solar reflector that was built on synthetic data. Masoum et. al. [10] performed a data-quality assessment of different saffron samples, employing data by second-order spectrophotometric-type, applying methods based on three-way chemometry. Fernández et al. [11] presented an estimation performed online of the electric arc furnace tap temperature.

Some mathematical modeling strategies of data-quality assessment are based on binary classification. DiFilippo [12] presented an assessment method that can be applied on SPECT and PET phantom images utilizing binary classification. Hoeijmakers [13] proposed an accuracy assessment of thermoacoustic instability models that involved binary classification.

Recent advances in neuroimaging research have begun to focus on the study of the interactions held at the level of brain regions. Garg et al. [14] proposed a causality analysis that is appropriate for fMRI data. The analysis, also called Granger causality, provides an efficient method for modeling the interactions treated at the spatiotemporal level that are held among the brain regions. The mentioned method applies to full-brain fMRI data.

Wang [15] proposes the use of Cohen’s Kappa, which is frequently applied in research as a quality measure for data annotations for binary classification tasks.

Saad et al. [16] performed research on improving the prediction power of the so-called chemometric models. This is realized through the exploitation of the supplied spectrophotometric data. The research also included a comprehensive bibliographic study. They mainly studied the outcome of data manipulation in the initial data-preprocessing step continuing with the application of chemometric models. Finally, the prediction power of the diverse models was matched by using a validation set consisting of eight mixtures. The statistical comparison was performed by using a two-factor Analysis of Variance. For additional comparison of the predictability of different built models, the prediction based on root-mean-squares error was compared.

### 2.2. State-of-the-Art Applications of Binary Logistic Regression

Applications of logistic regression are diverse, including processing of tomography [17], detection of rice seed purity [18], classification of scenes [19], spectral and spatial-based classification [20], prediction of axillary lymph node metastases [21], active smoking and associated behavioral risk factors before and during pregnancy [22]. BLR belongs to the class of logistic regression.

In the following, we present examples that illustrate the wide diversity of applications of BLR individually or in combination with other methods.

The applications in healthcare basically can be classified into two classes [23,24,25], namely prognostic prediction and diagnostic prediction. Prognostic prediction consists of the estimation of forming a particular disease throughout a specific period. Diagnostic prediction consists of the estimation of the probability of the presence of a considered disease. In the case of healthcare applications, we must also analyze the very important legal aspects. Reference [26] presents a valuable interdisciplinary study of the actual technical and legal challenges of data quality in the context of European medical law.

Saha et al. [27] treated the problem of predicting deforestation at the Gumani River Basin located in India. Cui et al. [28] presented research on driving forces of the urban hot spots. Barnieh et al. [29] studied the causal drivers of vegetation occurrence situated in West Africa. Ozen [30] presented research on damage gravity intensity assessment of pedestrian accidents. Sanchez-Varela et al. [31] presented research on the performing prediction of damage of status by applying procedures called active arranging training. Manoharan et al. [32] treated the problem of smart-grid checking by applying wireless devices. Lopez and Rodriguez [33] treated the problem of forecasting flash floods in Sao Paulo city. Gonzalez-Betancor and Dorta-Gonzalez [34] treated the subject of the danger of disruption of doctoral research and the problem of mental health in doctoral students.

Tesema et al. [35] presented performed research on trends of infant mortality in Ethiopia. The mathematical modeling was based on BLR with mixed-effect combined with analysis based on multivariate decomposition.

Ferencek and Borstnar [36] presented data-quality-assessment models applied for product failure prediction. The authors studied the failure prediction problem during the product warranty period. They proposed a machine-learning method that is able to decrease in time the error of prediction. From the initial 33 attributes, they detected seven with effective predicting power. One of the conclusions of the research was that the number of collected data was small associated with its cost.

Choi et al. [37] studied the problem of improving predictions efficiency by applying a method based on an artificial neural network resulting in a data-quality-assessment procedure. In the framework of a performed case study, the application to local scour across bridge piers was reported. The motivation of the performed research was based on the assumption of the authors that some methods based on artificial intelligence do not predict field-scale local scour with the necessary accuracy. The research included multivariate (methods applied: Euclidean distance and Mahalanobis distance) and univariate methods, and, finally, the obtained results are compared. The paper gives quantitative descriptions about the degree to how much the data-quality assessment improves predictions.

## 3. The Method Proposed for Data Quality Assessment

Approaches based on statistics are used for many real-life problem-solving situations [38,39,40,41]. Ref. [39] presented research focused on the study of the phytoremediation potential of crop plants. Regression analysis [42] is a statistical technique for estimating the strength of relationships among variables. Regression analysis includes the logistic regression that most frequently is applied in healthcare [43]. For instance, diverse medical scales for assessing the severity of a patient’s illness have been developed by using logistic regression [44,45,46,47].

Research regarding logistic regression involves one or more predictor (independent) variables and one predicted (dependent) variable. The logistic regression where the dependent variable is dichotomous (take two values) is called binary logistic regression (BLR), sometimes called binomial logistic regression. In our study, we consider the application of BLR. The BLR can be used with the purpose to check whether cases can be correctly predicted in two classes denoted as *ClassA* and *ClassB*. *ClassA* signifies the event occurring, for instance, an engine in a smart factory failed. *ClassB* signifies the event not occurring, for instance, an engine in a smart factory NOT failed. BLR calculates/estimates the probability of a case belonging to the specific *ClassA*. Let us denote with *P* the estimated probability of the event occurring. HA must establish a cut value denoted *CutV* that could take values in the interval [0, 1]. In most cases, the recommended *CutV* value is 0.5. If *p* ≥ *CutV*, the event is classified as occurring, and *ClassA* is chosen; elsewhere, if *p* < *CutV*, the event is classified as not occurring, and *ClassB* is chosen. BLR can predict whether cases can be accurately predicted/classified from the predictor variables.

Regarding the decision of choosing BLR alternatively with other methods, we must mention the decision that must be followed: When all predictors (can be one or more) are continuous, then a discriminant function analysis can be applied. When all the predictors (can be one or more) are categorical, a logit analysis can be applied. Logistic regression is appropriate even in the case when the predictor variables include both continuous and categorical types.

In the following subsections, we present assumptions that must be verified by using diverse statistical tests to be correctly applicable to the BLR. Moreover, we present the interpretations of the obtained results.

Logistic regression, in contrast to many statistic tests, makes no assumptions about the distributions of the independent variables. If it is useful for interpretation, a verification of normality assumption passing by the independent variables by scale type can be performed. For the verification of the passing of normality assumption for small sample size (3 to 50), we recommend the Shapiro–Wilk test (SW test) [48,49]. SW tests also have the advantage of having the highest statistical power and working well with few data [50]. For a large sample size, we recommend the Lilliefors test [51], which represents an adaptation of Kolmogorov–Smirnov test. Even though both tests can be applied at different significance levels (e.g., 0.01), we recommend the choosing, in most of the cases, the significance level *α_norm_* = 0.05, considering that it is the most appropriate. In the case of both tests, we also recommend the additional visual interpretation of normality by using the Quantile–Quantile plot (QQ plot) scatterplot [52]. In the case of normally distributed data, the points should fall approximately along this reference line. The greater the departure from the reference line, the greater the evidence is for the conclusion that the data fail the normality assumption.

### 3.1. Basic Assumptions

In this subsection, we present the basic assumptions denoted Basic Assumption 1 (BAss1), Basic Assumption 2 (BAss2), Basic Assumption 3 (BAss3) and Basic Assumption 4 (BAss4), which should pass before the application of BLR.

**Basic** **Assumption** **1.** *BAss1*

The predicted (dependent) variable should be measured on a dichotomous scale. For instance, in the case of a machine or sensor used in the industry, it could indicate the apparition or not of a failure. “C” could indicate correct functioning and “F” could indicate the apparition of failure.

**Basic** **Assumption** **2.** *BAss2*

The predictor (independent) variables (one or more) can take continuous or categorical values. Continuous values can be by interval or ratio type. Categorical values can be ordinal or nominal. The revision time of a machine or a sensor measured in time is an example of a continuous variable.

Ordinal variables can take, for instance, values on the Likert scale [53] by 3 points. For example, the defect of an engine or sensor is by a “small degree” (it can function even in the following), “noticeable” (it must be repaired) or “significant failure” (it is a very grave defect).

As examples of nominal variable values can be mentioned the quality of an engine or sensor that can be “Low” (low quality), “Moderate” (moderate quality), “High” (high quality) or “Excellent” (excellent quality).

In the following, we use the following notations: *np* denotes the number of predictor variables, and *n* denotes the total number of cases (data points). In an analysis, the number of valid cases can be smaller than the total number of cases, resulting from the elimination of invalid cases.

**Basic** **Assumption** **3.** *BAss3*

The predicted variable should have categories that are mutually exclusive and exhaustive.

**Basic** **Assumption** **4.** *BAss4*

This assumption states that should be verified for each continuous independent variable the existence of a linear relationship with the logit transformation of the dependent variable. The existence of the linearity can be made, for instance, using the Box–Tidwell method [54].

In the literature, there are proposed also some alternatives to the Box–Tidwell family of transformations to increase its generality. Reference [55] introduces fractional polynomials for Box–Tidwell transformations that have simple rational powers. Reference [56] presents another work on fractional polynomials.

**Basic** **Assumption** **5.** *BAss5*

Sample size estimation for BLR. An important factor for obtaining robust prediction performance is that the size of the dataset should be established relative to the number of predictors [57,58,59,60,61]. In the case of logistic regression, usually, the sample size is established as cases (events) per predictor variable in terms of events per variable (EPV). Low EPV values lead to poor predictive performance [58,60,62,63,64,65]. EPV is defined by the ratio of the number of events. EPV is calculated as the ratio of the number of events relative to the number of predictors.

In medical research, the rule of lower limit by EPV ≥ 10 is frequently used [66]. This criterion has a relatively general acceptance [24,67,68]. Some research [69,70,71] proved that this rule does not perform well in studies that involve large-scale simulation. References [63,65] proved that, when stepwise strategies for choosing predictor variables are applied, the criterion of EPV ≥ 10 is not appropriate. In such situations, it is necessary to apply the EPV ≥ 50 rule [63]. For the estimation of the predictive performance, the paper [72] considers the total sample size, the number of predictors and the events fraction.

### 3.2. Assumptions That Must Be Passed by the BLR

In this subsection, we present the assumptions, which should be verified by using statistical tests, whose results must pass the threshold values established by HA. If at least one of these assumptions fail, then the application of the BLR is not correct. There are also presented some other statistical tests that must be applied and whose results must be interpreted.

#### 3.2.1. Test for Variables in the Equation Considering the Intercept Only Model

Table 1 presents the study performed for the variables in the equation considering the intercept-only (constant) model. With this purpose is applied Wald’s Chi-Square test. Essential aspects regarding Wald’s test in References [73,74] are presented. In Table 1, we use the following notations: *ValWald* represents the obtained value of Wald’s test statistics; *Valβ* represents the value of the obtained *β*; *ValSe* represents the value of the standard error; *ValSigVE* represents the *p*-value of the Wald’s test; and *Exp*(*β*) represents the odds ratio predicted by the model, [*Exp*(*β*)] represents the predicted odds of deciding on *ClassA*, [*Exp*(*β*)] *= ValExpβ*.

In the case of the assumption of Wald’s test, as applied for variables in the equation, it should pass. This means that we must obtain a result for *Sig_ve_* that is statistically significant. Moreover, *α_ve_* denotes the significance level of Wald’s test. We recommend that the *α_ve_* value be 0.05. For the *Sig_ve_* value, *Sig_ve_* < *α_ve_* indicates significance.

Based on Table 1 the intercept-only model can be formulated as (1)

ln(odds) = *Valβ*, (1)

#### 3.2.2. Omnibus Test of Model Coefficients

When checking the fact that the model is an improvement over the baseline model, we must apply the Omnibus Test of Model Coefficients (OT). It is considered the model with included explanatory variables. OT applies Chi-Square tests for the verification if is a considerable difference between the Log-likelihoods (-2LLs) of the new model and the baseline model. If the model has a significantly increased -2LL comparatively with the baseline, this shows that the new model is explaining more of the variance in the predicted variable, thus indicating the obtainment of an improvement. Moreover, α_o_ denotes the applied significance level of the OT that must be set by HA. The recommended value for α_o_ is 0.01 in most of the cases.

**Assumption** **1** **(Ass1).** *Result of Omnibus Test of Model Coefficients.*

By applying the OT, we should obtain a statistically significant Chi-Square value. *Sig_ot_* denotes the obtained *p*-value of the OT. An *Sig_ot_* < α_o_ indicates the appropriateness of the application of BLR.

There are three different versions of OT, namely Model, Step and Block. For the comparison of the new model (full model) to the baseline (no model), we should use the version, called Model. The null hypothesis (H_0_) states that the full model and the baseline model are not statistically significantly different. The rejection of H_0_ leads to acceptance of alternative hypothesis H_1_ that allows the formulation of the conclusion that the full model and the baseline model are statistically significantly different. If we apply a stepwise procedure of adding explanatory variables to the model hierarchically, then we should be using the Step and Block. 

#### 3.2.3. Hosmer–Lemeshow Test

Hosmer and Lemeshow [75] proposed a goodness-of-fit-test called the Hosmer–Lemeshow (HL) test that is an assumption test for logistic regression models. HL statistic is a reliability indicator of model fit for BLR. This test admits determining whether the model adequately describes the data, and α*_hl_* denotes the applied significance level of the HL test that should be set by HA. The recommended value for α*_hl_* is 0.05 in most of the situations. *Sig_hl_* indicates the significance level obtained by applying the HL.

**Assumption** **(Ass2).** *Hosmer–Lemeshow test result.*

For the BLR to correct applicability, it should verify the criterion of non-significance of the Hosmer–Lemeshow test, *Sig_hl_* > α*_hl_*. The non-significance is an indicator of a good model fit. A significance value, *Sig_hl_*, lower than 0.05 indicates poor fit and indicates the non-appropriateness of the application of BLR. 

#### 3.2.4. The Variance Explained in the Dependent Variable

In this subsection, we address the subject of testing how much variance in the predicted variable can be explained by using the model. More concretely, we present the statistics that can be performed and the interpretation of the results. We also address Assumption 3 (Ass3), which should be verified and passed.

The coefficient of determination in multiple regression analysis is denoted by R^2^. R^2^ takes values in the interval [0, 1] and determines the amount of variance in the dependent variable that can be explained by the predictor variables.

In the case of BLR, we should evaluate the pseudo-R^2^ [76]. As an observation, we would like to mention that the pseudo-R^2^ values usually have lower values than R^2^ values in multiple regression. There are different methods proposed in the scientific literature for calculating the pseudo-R^2^ [76], without having a unanimous agreement on which of them is the most appropriate. In the case of each of them, we can consider advantages and disadvantages. With this purpose, to obtain the explained variance, we can use the likelihood ratio R^2^ proposed by the Cohen [76], Cox and Snell R^2^ [77,78], McFadden R^2^ [78], Tjur R^2^ [78], and Nagelkerke R^2^ [76] tests. Tjur R^2^ is relatively new.

Reference [78] presents a limitation of the Cox and Snell R^2^. Cox and Snell R^2^ cannot reach the value 1. The Nagelkerke R^2^ test provides a correction of the Cox and Snell R^2^. Among others, the correction includes the elimination of the weakness of not reaching the value 1. The different calculus performed by these two tests provides different results. Reference [78] proposes the use of McFadden R^2^ instead of Cox and Snell R^2^. This is based on the different scaling of the tests. In the following, we suggest for interpretation the Nagelkerke R^2^ value. An obtained NrS value of pseudo-R^2^ indicates that around NrS% of variance in the dependent variable can be explained by the predictor variables.

**Assumption** **3** **(Ass3).** *Minimal variance in the dependent variable that should be explained.*

In the case of an experimental evaluation, HA could establish classes of prediction power as follows: “unsatisfactory”, “week”, “appropriate”, “good” and “very good”. Table 2 presents such a proposed classification that could be applied in most of the cases. HA must establish the minimal class to which should be expectable to belong the prediction power of the variables. For instance, HA could establish that it is expectable to belong to at least the class of “good” prediction power.

**Assumption** **4** **(Ass4).** *Measuring how poor is the prediction power*.

Additionally, we can calculate the so-called -2 Log-likelihood statistic [79], which represents a measure of how poor the prediction power is. A smaller value of the statistic indicates a better model. The value considered by itself does not provide a clear interpretation. It is useful when more models are compared based on the value of the Log-likelihood. This can be used, for instance, when testing the predicting power of different sets of predictor variables. 

#### 3.2.5. Evaluation of Obtained Classification

The results obtained by applying the BLR can be used for making a binary classification. In the following, we consider the assessment of the effectiveness of the predicted classification. It is necessary to assess the effectiveness of the known (correct) classification against the predicted classification. For this, we suggest as a first step the construction of a so-called classification table (CT). Table 3 presents the structure of a CT, consisting of the observed and predicted binary classification. As an example of applicability can be mentioned the machine failure (MF) prediction (a machine fails or not).

In the following is presented the calculus of some indicators that admit the comparison of classification results of BLR with the results of other methods used with the same classification purposes. TN denotes the true negatives: negatives that were correctly classified, TN = *Val1*. TP denotes the true positives: positives that were correctly classified, TP = *Val4*. FP denotes the false positives: values that are classified as YES (positives) but are NO (negatives), FP = *Val2*. FN denotes the false negative: values that are classified as NO (negatives) but are YES (positives), FN = *Val3*.

Based on the values retained in Table 3, we recommend the calculus of the principal indicators that we consider: sensitivity, specificity and accuracy.

Sensitivity, denoted as *ValSENS%* in the table, represents the percentage of the cases that had the observed characteristic (“YES” for machine failure); the cases were correctly predicted by the model.

*ValSENS%* = 100 × (TP/(TP + FN)) = 100 × (*Val4*/(*Val4* + *Val3*));

Specificity, denoted as *ValSPEC%* in the table, represents the percentage of cases that did not have the observed characteristic (“NO” for machine failure); these cases were correctly predicted as not having the observed characteristic.

*ValSPEC%* = 100 × (TN/(TN + FP)) = 100 × (*Val1*/(*Val1* + *Val2*));

Accuracy represents the percent of correctly predicted positives and negatives. *ValTot%* from Table 2 denotes the accuracy.

*ValTot%* = 100 × ((TN + TP)/(TN + TP + FN + FP)) = 100 × ((*Val1* + *Val4*)/(*Val1* + *Val4* + *Val2* + *Val3*));

**Assumption** **5** **(Ass5).** *Classification results evaluation*.

Performing the classification and considering the specificity of the research, we should obtain at least a threshold minimal admitted value for one of the following:

- The accuracy indicator;
- All the indicators accuracy, sensitivity and specificity.

This must be established by the human assessor.

Comparing the obtained indicator value(s) with the required one, we can establish the threshold values determined by HA if the classification is in accordance with the expectations. These indicator values admit the comparison with results of other models, based on the fact that HA can choose the most appropriate one. If necessary, HA can choose some other well-known indicators, such as the F1 score [80], AUC [81], ROC [82], TSS [83] and Kappa coefficient [84].

#### 3.2.6. Variables in the Equation with Predictor Variables Included

As a following step, it is applied the Wald Chi-Square test [73,74]. Wald’s test is used for testing the individual contribution of each predictor variable (one or more predictors). Each predictor variable is analyzed in the context of all other predictor variables whose values are kept constant. This procedure has the advantage of being able to eliminate the overlaps between the predictor variables. In the case of a predictor variable, we must verify if it is a statistically significant predictor. It must be applied for each predictor variable at the *α_Sig_* significance level, which must be set by HA. The significance level, *α_Sig_*, could take different values, such as 0.05, 0.01 and 0.001. We consider that, in many cases, the most appropriate value is 0.05. In the case of each of the included predictor variables, a value where *Sig_ve_* < *α_Sig_* indicates that the variable is a significant predictor. Elsewhere, if in case of a predictor variable, *Sig_ve_* ≥ *α_Sig_* indicates that the variable is NOT a significant predictor. 

Table 4 presents the variables in the equation with the predictor variables included. The columns in Table 4 have the same labeling as those in Table 1. In Table 1, we use the following notations: *β* denotes regression weight in the model, *SE* denotes the standard error, *WaldStat* denotes the Wald’s test statistics (Wald’s Z value) and *EXP(β)* denotes the odds ratio (OR). The power of the association between two events can be measured by using the OR.

For explanatory purposes, we present fictitious data in Table 4 as results regarding the predictor variables in the regression equation. *Var*_1_(CatA), *Var*_2_ and *Var*_3_ denote the predictor variables, where *Var*_1_ is a categorical variable, with CatA considered as reference category. *Var*_2_ and *Var*_3_ take continuous values. The regression coefficients denoted β gives the predicted amount of change in the dependent. *Exp(β)* is the odds ratio (OR) predicted by the model. OR is calculated by raising the base of the natural Log to the sth power, s denotes the slope that results from the logistic regression equation and CI of *EXP(β)* denotes the confidence interval of *EXP(β)* that must be set by HA. It is recommended to choose the 95% confidence level. *LowExpβ* represents the lower limit of the confidence interval, and *UppExpβ* represents the upper limit of the confidence interval. 

In the following, for illustrative purposes, we interpret the fictitious data from Table 4. Considering *Var*_1_, based on the fact that *Sig_ve_* < *α_Sig_* (0.01 < 0.05), we can conclude that *Var*_1_ is a significant predictor. Considering *Var*_2_, based on the fact that *Sig_ve_* < *α_Sig_* (0.03 < 0.05), we can conclude that *Var*_2_ is a significant predictor. Considering *Var*_3_, based on the fact that *Sig_ve_* > *α_Sig_* (0.83 > 0.05), we can conclude that *Var*_3_ it is NOT a significant predictor.

*Exp(β)* of Table 4 includes the information for predicting the probability of an event occurring when all other predictor variables are kept constant. For example, in the case of *Var*_1_, the table shows that the odds for the “Yes” category are 22.65 times greater for CatA as opposed to CatB.

The *Sig_ve_* value in the case of each predictor variable gives an answer if the variable is a significant predictor or not. If there are variables that are not significant predictors, this can be reported in the formulated conclusions. If decided, they can be removed, and the analysis can be repeated.

The variables in the equation output obtained for the fictitious data give us the following regression equation, Equation (2):

ln(odds) = −37.7 + 0.78 × *Var*_1_ − 0.75 × *Var*_2_ + 0.02 × *Var*_3_, (2)

The obtained model can be used to predict the odds, based on Equation (3).

(3) $$\mathrm{odds}=e^{\left(-37.7\;+\;0.78\;\times \;Var_{1}\;-\;0.75\;\times \;Var_{2}\;+\;0.02\;\times \;Var_{3}\right)},$$

Conversion to probabilities should be realized as follows (4):

(4) $$P=\frac{\mathrm{odds}}{1+\mathrm{odds}}\;.$$

If decided, we can apply an approach whereby each predictor variable is eliminated step-by-step from the model and is compared to the −2 Log-likelihood statistic of the reduced model with the full model to establish if the reduction is appropriate. Let us consider *n* to be the number of predictor variables. The previously mentioned approach requires testing of *n +* 1 models.

### 3.3. Interpretation of the Binary Logistic Regression Results

An iterative maximum likelihood procedure constructs the model. It starts with values of the regression coefficients generated arbitrarily based on that the construction of an initial model that predicts the observed data. After this, the errors are evaluated and, based on this, the regression coefficients change. This is performed in such a way as to make the observed data likelihood superior for the novel model. This procedure is repeated recursively until the convergence of the model is reached.

In Section 3.1 and Section 3.2, we present all the assumptions that must be passed for the application of BLR to be correct. In this section, we present the interpretation of the obtained results in case of passing all the mandatory assumptions.

In Section 3.2.4, we present the calculus of the pseudo-R^2^, whose result is denoted NrS. HA considering the specificity of the research must create the interval classes for the value of pseudo-R^2^ that must be labeled, for instance, in Table 2: “unsatisfactory”, “week”, “appropriate”, “good” and “very good”.

Section 3.2.5 presents the performed classification results in the form of a classification table. Moreover, there are recommended indicators accuracy, sensitivity and specificity. HA considering the specific of the research must establish the thresholds (threshold, minimal acceptance values) for the indicators values that are considered in the interpretation of the results.

## 4. Experimental Data-Quality-Assessment Evaluation

### 4.1. The Synthetic Dataset Used in the Evaluation

UCI is a valuable repository of datasets that can be used for research purposes [85]. Reference [1] provides a synthetic dataset created with the intent to mimic real industrial predictive maintenance data. In this section, we briefly present the description of the UCI dataset that we used in the experimental data-quality-assessment evaluation. Reference [4] presents the dataset’s details. Table 5 presents a snapshot of the data with included three cases (numbered with 1, 2 and 78). The whole dataset includes 10,000 rows (cases) of data characterized by six features/variables, organized on separate columns in the table, in the case of real-life data provided by different sensors (e.g., intelligent sensor camera, thermistors, etc.). The features, independent (predictor) variables, are as follows: *V*_1_, the product ID; *V*_2_, the air temperature (K); *V*_3_, the process temperature (K); *V*_4_, the rotational speed (rpm); *V*_5_, the torque (Nm); and *V*_6_, the tool wear (min). Where K denotes the Kelvin temperature scale where zero reflects the complete absence of thermal energy, rpm denotes rotations per minute; Nm denotes the Newton-meter that is a unit of torque in the SI system and min denotes the minutes.

*V*_1_ values are labeled with the letters “L”, “M” and “H” based on the quality of the product. “L” denotes low-quality products, which consist of 50% of all products. “M” denotes medium quality products, which consist of 30% of all products. “H” denotes high-quality products, which consist of 20% of all products.

Karl Pearson first defined the term “random walk” [86]. Data retained in *V*_2_ were generated by utilizing a random-walk process that is later normalized to a standard deviation of 2K around 300K. Data retained in *V*_3_ were generated by utilizing a random-walk process that was normalized to a standard deviation of 1K, added to the air temperature plus 10K. Data retained in *V*_4_ were calculated from a power of 2860 W, which was overlaid with noise that was normally distributed. Data retained in *V*_5_ consisted of the torque values that are normally distributed around 40 Nm with a standard distribution of σ = 10 Nm (note that there are no negative values). Data retained in *V*_6_ represent the quality variants H/M/L add 5/3/2 minutes of tool wear to the used tool in the process. 

The dependent variable *V_F_* is labeled “machine failure”. The retained values indicate whether the machine has failed in a different particular data point. The value 0 indicates no failure. The value 1 indicates the apparition of a failure.

### 4.2. Experimental Evaluation Results

**Figure 1:**
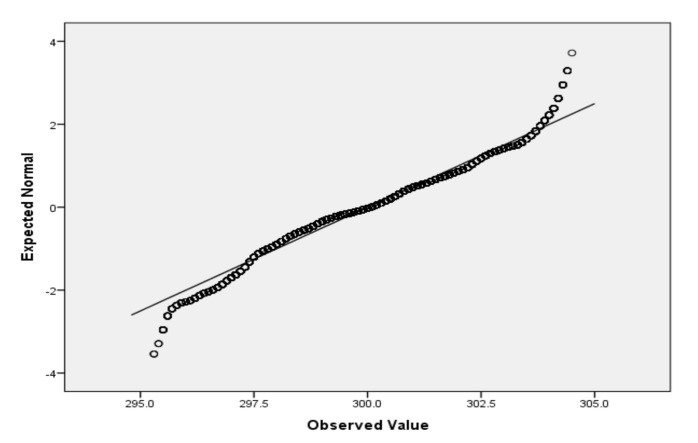
QQ plot for *V*_2_.

**Figure 2:**
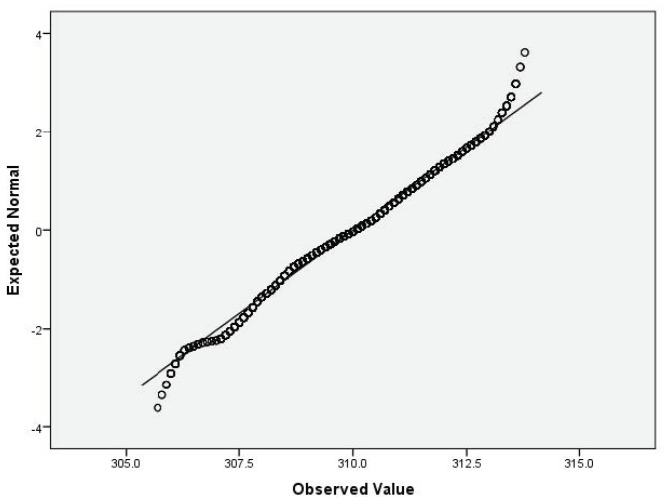
QQ plot for *V*_3_.

**Figure 3:**
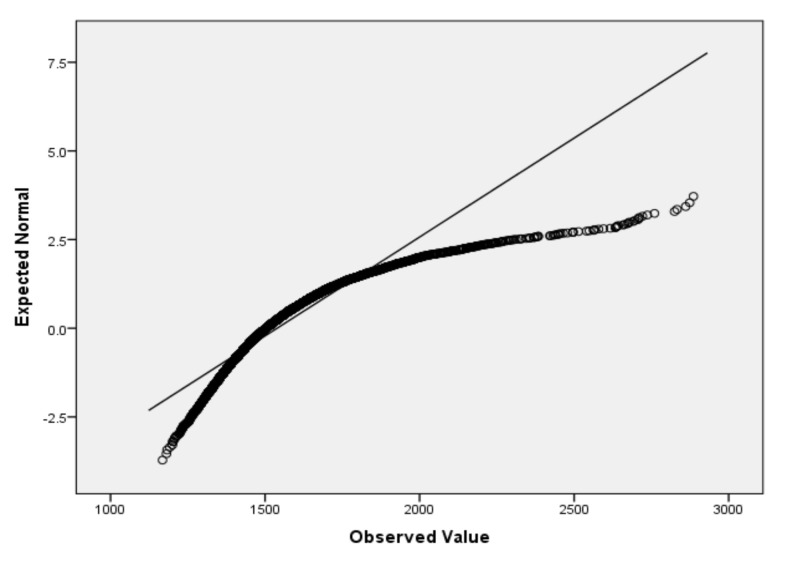
QQ plot for *V*_4_.

**Figure 4:**
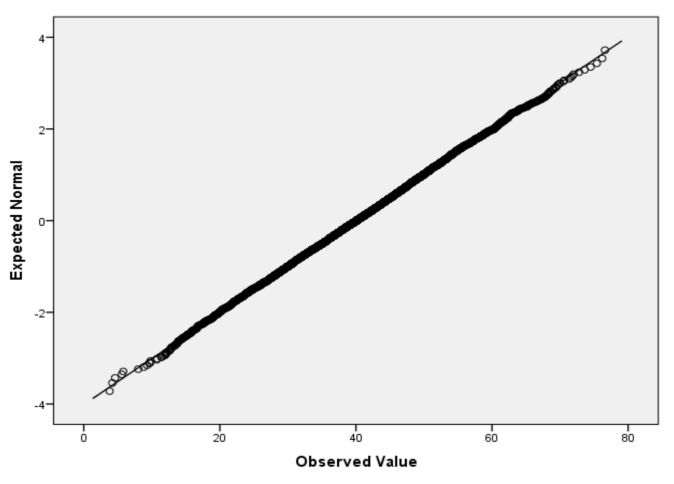
QQ plot for *V*_5_.

**Figure 5:**
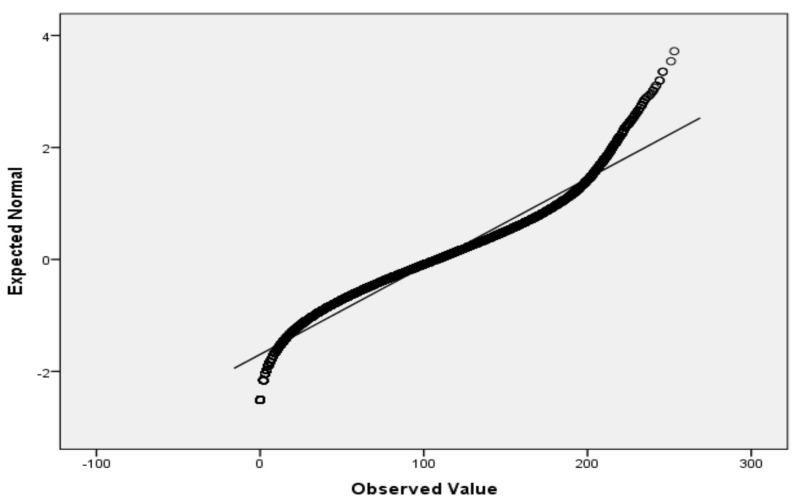
QQ plot for *V*_6_.

For the variables *V*_2_ (air temperature), *V*_3_ (process temperature), *V*_4_ (rotational speed), *V*_5_ (torque) and *V*_6_ (tool wear), based on the large sample size, we applied the Lill test with the significance level α_norm_ = 0.05 (Table 6) and created the QQ plots (Figure 1, Figure 2, Figure 3, Figure 4 and Figure 5) for visual interpretation. The degrees of freedom of each variable is 10,000.

We performed a BLR on the data presented in Section 4.1. All the cases, 100%, were included in the study, since they were valid (do not contains missing data and outliers). In the experimental evaluation study, we used the following predictor variables: *V*_2_ (air temperature), *V*_3_ (process temperature), *V*_4_ (rotational speed), *V*_5_ (torque) and *V*_6_ (tool wear).

For the interpretation of the results, we must mention that, in the following, the predicted probability is for the membership for 1 that denotes failure.

Step 1. Verification of passing all the basic assumptions.

All the basic assumptions, BAss1, BAss2, BAss3, BAss4 and BAss5, received verification that they passed the HA.

Step 2. Performing the test for the variables in the equation considering the intercept-only model.

Table 7 presents the Wald test results regarding the variables in the equation considering the intercept-only model. The significance level, α*_ve_*, was set to the value 0.05 by HA. The predicted odds of deciding to continue the research are odds [*Exp(B)*] = 0.035, which is relatively low. However, the criterion of the significance of variables in the equation passed, *Sig_ve_* ~ 0, *Sig_ve_* < *α_ve_*, indicating a significant result.

Step 3. Performing the Omnibus Test of Model Coefficients

In the following, we applied the Omnibus Test of Model Coefficients. The value of significance level *α_ot_* was set to 0.01 by HA. Table 8 presents the results of the OT. According to the previous explanations, it should be interpreted just the line labeled “Model” with the Chi-Square value 1038.064 and *Sig_ot_ =* ~0. The obtained result, *Sig_ot_*, where *Sig_ot_* < *α_ot_*, indicates significant result. However, Assumption Ass1 passed.

Step 4. Performing the Hosmer–Lemeshow Test

We performed the Hosmer–Lemeshow test. The significance level of the HL, the α*_hl_* value, was set to 0.05 by HA. Table 9 presents the results of the HL. Since the *p*-value of the HL is *Sig_hl_* > α*_hl_*, we can conclude that the criterion of non-significance of HL is satisfied, as is the requirement for the correct application of BLR. Henceforth, we show the good fit of the model. However, assumption Ass2 passed.

Step 5: The variance explained in the dependent variable

Regarding the value of pseudo-R^2^ based on the specific of the research, HA makes the classification that was presented in Table 1. At this step, we calculated the pseudo-R^2^ by using two methods. Table 10 presents the obtained results of the Cox and Snell R^2^ and Nagelkerke R^2^ values. Cox and Snell R^2^ = 0.099, and Nagelkerke R^2^ = 0.385. 

For the interpretation, we used the Nagelkerke R^2^ = 0.385 based on motivations previously explained. This obtained value indicates that almost 39% of the variance in the predicted variable can be explained by the predictor variables *V*_2_, *V*_3_, *V*_4_, *V*_5_ and *V*_6_. The classification results presented in Table 10 indicate a prediction power, which is classified as “good” according to Table 1, 39% ∈ (30%, 40%). However, Ass3 passed. The value of −2 Log-likelihood, Ass4, can be used with comparative purposes to decide which of predictor variables should be maintain, considering their predictive power.

Step 6. Performing the classification by using the binary logistic regression.

HA established that the classification accuracy must belong to the interval (95%, 100%). Table 11 presents the results of classification obtained by applying BLR.

The results presented in Table 11 indicate the classification accuracy by 97%, 97%∈[95%, 100%], which, based on the threshold established by HA, fits the expectation. However, Ass5 passed. It should be mentioned that, in the classification, we used the cut value 0.5. The value of the additionally calculated specificity is *ValSPEC%* = 99.7%. The value of the additional calculated sensitivity is *ValSENS%* = 20.1%.

Step 7. Performing the Wald’s test for the variables in equation with the predictor variables included.

We performed the Wald’s test for the variables in the equation. Table 12 presents the results of Wald’s test, as performed on the variables *V*_2_ (air temperature), *V*_3_ (process temperature), *V*_4_ (rotational speed), *V*_5_ (torque) and *V*_6_ (tool wear). HA establishes the significance level as *α*_Sig_ = 0.05.

For each of the predictor variables, namely *V*_2_, *V*_3_, *V*_4_, *V*_5_ and *V*_6_, the Wald’s test result *Sig_ve_* < α_Sig_ indicates that the variable is a significant predictor. It is considered the confidence interval, [*LowExpβ*, *UppExpβ*] of *EXP(β)* at the CI = 95% confidence level.

Moreover, we must note the following:

For *V*_2_, *Exp(β* *) >* 1 and *LowExpβ* *>* 1, *UppExpβ* *>* 1.

For *V*_3_, *Exp(β) <* 1 and *LowExpβ* *<* 1, *UppExpβ* *<* 1.

For *V*_4_, *Exp(β) >* 1 and *LowExpβ* *>* 1, *UppExpβ* *>* 1.

For *V*_5_, *Exp(β) >* 1 and *LowExpβ* *>* 1, *UppExpβ* *>* 1.

For *V*_6_, *Exp(β) >* 1 and *LowExpβ* *>* 1, *UppExpβ* *>* 1. 

The regression equation can be formulated based on the results presented in Table 11 as follows (5):

ln(odds)= −36.69 + 0.772 × *V*_2_ − 0.743 × *V*_3_ + 0.012 × *V*_4_ + 0.281 × *V*_5_ + 0.013 × *V*_6_, (5)

The obtained model can be used to predict the odds, based on Equation (6).

(6) $$\mathrm{odds}=e^{-36.69\;+\;0.772\;\times \;V_{2}\;-\;0.743\;\times \;V_{3}\;+\;0.012\;\times \;V_{4}\;+\;0.281\;\times \;V_{5}\;+\;0.013\;\times \;V_{6}},$$

Conversion to probabilities should be realized as follows (7):

(7) $$P=\frac{\mathrm{odds}}{1+\mathrm{odds}}$$

Step 8. Improving the classification by adjusting the cut value.

Regarding the classification choosing the cut value 0.4, the obtained results are presented in Table 13. The accuracy is 97.1% (97% for the cut value 0.5). The value of specificity is *ValSPEC%* = 99.5% (99.7%, for cut the value 0.5). The value of sensitivity is *ValSENS%* = 28.3% (20.1% for the cut value 0.5). The obtained results for the cut value 0.4 are better than those obtained for the cut value 0.5.

## 5. Discussion

Initially, we included in the model the predictor variable *V*_1_, along with *V*_2_, *V*_3_, *V*_4_, *V*_5_ and *V*_6_. The −2 Log-likelihood obtained was 1913.929. Wald’s test for the variables in the equation with *Sig* = 0.69 indicated the fact that *V*_1_ is not a significant predictor. Based on this fact, *V*_1_ was removed from the second experimental setup presented in Section 4.2. When verifying the results of the classification table, we observed that we obtained better results with *V*_1_ removed. The necessity of a new model was motivated also by the result of the HL test that indicated non-significance.

Briefly, the obtained results presented in the previous section can be interpreted as follows. For the studied synthetic data-quality assessment [1], we applied a BLR. In the case of BLR, we verified the passing of all the necessary assumptions for its applicability. The obtained results indicate the fact that the synthetic data passed the data-quality assessment, indicating that the predicting power is enough to be applicable to binary prediction/classification algorithms. Finally, we presented the classification results based on the applied model, with the cut value 0.4 presented in the form of a classification table, which admits the clear comparison of the results with the prospective results of other methods that can be applied. Based on the value from the classification table, we calculated the accuracy, sensitivity and specificity, but we can use any other indicators, if necessary, such as the F1 score [80], ROC [82], area under the ROC curve (AUC) [81], TSS [83] and Kappa coefficient [84].

The errors that could appear at a classification are the false positives and false negatives. It must be noticed that, in the classification tests, ROC and AUC present how an adjustable threshold causes changes in two types of errors. However, the ROC curve and AUC are only partially meaningful when used with unbalanced data. To avoid this problem, Reference [87] introduced a new concordant partial AUC and a new partial c statistic for ROC.

Regarding the interpretation of the research results, we can say that a BLR was performed to ascertain the effects of *V*_2_ (air temperature), *V*_3_ (process temperature), *V*_4_ (rotational speed), *V*_5_ (torque) and *V*_6_ (tool wear) on the likelihood of the machine failure. All the assumptions necessary for the application of BLR passed. The applied model explained 38.5% (chosen the Nagelkerke pseudo-R^2^) of the variance in machine failure. The model correctly classified 97.1% of the cases (with the chosen cut 0.4). Increasing *V*_2_ was associated with an increased likelihood of machine failure. Similarly, increasing *V*_4_, *V*_5_ and *V*_6_ were associated with an increased likelihood of machine failure, but the likelihood of failure is smaller than for *V*_2_. On the other hand, increasing *V*_3_ was associated with a reduction in the likelihood of exhibiting machine failure.

## 6. Conclusions

In Industry 4.0 and smart factories, there are many challenging binary prediction and classification types of problems that should be solved. Frequently obtaining real industrial data is difficult or even impossible. Based on this fact, very often we should use synthetic data obtained as a result of real or software simulations. In the case of such data, a fundamental subject that should be treated is making a data-quality assessment. We consider that one of the most important aspects of data-quality assessment that should be evaluated is the predictive power. The passing of the data of all the indicated assumptions is an indication for researchers that, on the data, can be applied even other algorithms for binary prediction (classification), offering, at the same time, a comparison measure that is possible to be passed by other algorithms.

The data-quality-assessment fundament was the first objective of the research. For this data-quality-assessment verification, we indicated that a BLR should be performed. By applying this method is obtained the response to the model fits the data. On the obtained classification table, which practically consists of the confusion matrix, we applied the indicators accuracy, sensitivity and specificity. Some others are also recommended to be used when considered appropriate.

BLR is frequently applied in research for solving a binary classification problem and the prediction of the membership to a specific class with a certain probability. Its applicability is best known in medical research. In the case of its application, frequent mistakes result from the fact that the assumptions that should pass to correct its application are not verified. Such mistakes could lead to formulating erroneous conclusions of the research. The second objective of the paper was in making the mathematical grounding of the assumptions that should be verified at the application of the binary logistic regression. These assumptions make it possible for the researchers to apply BLR correctly and correctly interpret the obtained results.

## Figures and Tables

**Table 1:** Variables in the equation considering the intercept-only model.

	*β*	*SE*	*WaldStat*	*Sig_ve_*	*Exp(β)*
Constant	*Val* *β*	*ValSe*	*ValWald*	*ValSigVE*	*ValExpB*

**Table 2:** Prediction power classification established by HA.

Name of the Class	Interval
“unsatisfactory” prediction power	<10%
“week” prediction power	(10%, 20%)
“appropriate” prediction power	(20%, 30%)
“good” prediction power	(30%, 40%)
“very good” prediction power	≥40%

**Table 3:** Structure of classification table for machine-failure prediction.

		Predicted		
		Machine Failure		Percentage Correct
		NO	YES	
**Known**	**NO**	*Val1*(TN)	*Val2*(FP)	*ValSPEC%*
	**YES**	*Val3*(FN)	*Val4*(TP)	*ValSENS%*
**Accuracy**				*ValTot%*

**Table 4:** Variables in the equation with predictor variables included.

	*β*	*SE*	*WaldStat*	*Sig_ve_*	*Exp(* *β* *)*	CI of *EXP(β)*	
						*LowExp* *β*	*UppExp* *β*
*Var*_1_(CatA)	0.78	0.072	114.079	0.01	22.65	11.879	22.495
*Var*_2_	−0.75	0.096	59.442	0.03	0.476	0.394	0.575
*Var*_3_	0.02	0.001	473.247	0.83	1.012	1.011	1.013
Constant	−37.7	14.641	6.278	0.012	0		

**Table 5:** Snapshot of the data used in the research.

UDI	Product ID	*V*_1_	*V*_2_	*V*_3_	*V*_4_	*V*_5_	*V*_6_	*V_F_*
1	M14860	M	298.1	308.6	1551	42.8	0	0
2	L47181	L	298.2	308.7	1408	46.3	3	0
…	…	…	…	…	…	…	…	…
78	L47257	L	298.8	308.9	1455	41.3	208	1
…	…	…	…	…	…	…	…	…

**Table 6:** Results of the data normality verification.

	*V*_2_	*V*_3_	*V*_4_	*V*_5_	*V*_6_
Statistic	0.067	0.49	0.104	0.009	0.06
*p*-value	0	0	0	0.64	0
QQ plot	Figure 1	Figure 2	Figure 3	Figure 4	Figure 5
Normality assumption passing (*p*-value > α_norm_)	No	No	No	Yes	No

**Table 7:** Variables in the equation considering the intercept-only model.

	*β*	*SE*	*Wald*	*Sig_ve_*	*Exp(* *β* *)*
Constant	−3.35	0.055	3675.133	~0	0.035

**Table 8:** Result of the Omnibus Test of Model Coefficients.

	*Chi-Square*	*Sig_ot_*
Step	1038.064	~0
Block	1038.064	~0
Model	1038.064	~0

**Table 9:** Result of the Hosmer–Lemeshow test.

*Chi-Square*	*SigHL*
13.39	0.099

**Table 10:** Obtained pseudo-R^2^ values.

−2 Log-Likelihood	Cox and Snell R^2^	Nagelkerke R^2^
1922.894	0.099	0.385

**Table 11:** Performed classification results, with the cut value set to 0.5.

Known	Predicted		
	Machine Failure		Percentage Correct
	NO Failure (0)	Failure (1)	
NO Failure (0)	9635	26	99.7%
Failure (1)	271	68	20.1%
Accuracy			97.0%

**Table 12:** Variables in the equation with the predictor variables included.

	*β*	*SE*	*WaldStat*	*Sig_ve_*	*Exp(β)*	CI of *EXP(β)*	
						*LowExp* *β*	*UppExp* *β*
*V*_2_	0.772	0.072	114.079	~0	2.165	1.879	2.495
*V*_3_	−0.743	0.096	59.442	~0	0.476	0.394	0.575
*V*_4_	0.012	0.001	473.247	~0	1.012	1.011	1.013
*V*_5_	0.281	0.011	599.492	~0	1.324	1.295	1.354
*V*_6_	0.013	0.001	138.315	~0	1.013	1.011	1.016
Constant	−36.69	14.641	6.278	0.012	0		

**Table 13:** Performed classification results from using the method with the cut value 0.4.

Known Machine Failure		Predicted		
		Machine Failure		Percentage Correct
		0	1	
NO Failure	0	9609	52	99.5%
Failure	1	243	96	28.3%
Accuracy				97.1%

## Data Availability

We used publicly available data for research on UCI data repository [2].
